# Gender and Advanced Urothelial Cancer: Outcome, Efficacy and Toxicity following Chemotherapy

**DOI:** 10.3390/medicina58070886

**Published:** 2022-07-01

**Authors:** Lucrezia Becattini, Calogero Saieva, Laura Doni, Giandomenico Roviello, Pietro Spatafora, Martina Catalano, Francesco Sessa, Ilaria Camilla Galli, Claudio Bisegna, Francesco Lupo Conte, Claudia Zaccaro, Raffaella Santi, Sergio Serni, Gabriella Nesi, Donata Villari

**Affiliations:** 1 School of Human Health Sciences, University of Florence, 50134 Florence, Italy; lu.becattini@gmail.com (L.B.); martina.catalano@unifi.it (M.C.); claudio.bisegna@unifi.it (C.B.); francescolupo.conte@unifi.it (F.L.C.); claudia.zaccaro@unifi.it (C.Z.); 2 Cancer Risk Factors and LifeStyle Epidemiology Unit, Institute for Cancer Research, Prevention and Clinical Network (ISPRO), 50139 Florence, Italy; c.saieva@ispro.toscana.it; 3 Department of Medical Oncology, Careggi Teaching Hospital, 50134 Florence, Italy; donila@aou-careggi.toscana.it; 4 Department of Health Sciences, University of Florence, 50139 Florence, Italy; giandomenico.roviello@unifi.it (G.R.); raffaella.santi@unifi.it (R.S.); 5 Unit of Urological Minimally Invasive Robotic Surgery and Renal Transplantation, Careggi Teaching Hospital, 50134 Florence, Italy; spataforap@aou-careggi.toscana.it (P.S.); sessaf@aou-careggi.toscana.it (F.S.); 6 Histopathology and Molecular Diagnostics, Careggi Teaching Hospital, 50139 Florence, Italy; galliic@aou-careggi.toscana.it; 7 Department of Experimental and Clinical Medicine, University of Florence, 50134 Florence, Italy; sergio.serni@unifi.it (S.S.); donata.villari@unifi.it (D.V.)

**Keywords:** muscle-invasive bladder cancer, upper urinary tract urothelial carcinoma, chemotherapy, gender, oncological outcome, toxicity

## Abstract

*Background and Objectives*: The incidence of urothelial cancer in males is higher than in females; however, females have a higher risk of recurrence and progression. The aim of our study was to report the effect of gender on the oncological outcome in advanced urothelial cancer. *Materials and Methods*: In our retrospective study, all patients had undergone primary surgical treatment for urothelial cancer and were affected by stage IV disease at the time of chemotherapy. Response to therapy and toxicity were evaluated. Subgroups were analyzed for tumour presentation, first- and second-line treatment response, progression-free survival (PFS) and overall survival (OS). *Results.* Seventy-five patients, 18 (24%) females and 57 (76%) males, were considered. Investigation into the distribution of individual characteristics according to gender revealed a significant difference only for smoking, with a prevalence of smokers in women (*p* = 0.029). At the end of follow-up, OS was higher in females (27.5% vs. 17.4%; *p* = 0.047). Smoking did not significantly influence OS (*p* = 0.055), while univariate Cox regression analysis confirmed that males had a higher risk of death (HR = 2.28, 95% CI 0.99–129 5.25), with borderline statistical significance (*p* = 0.053). Men showed higher PFS than women both after first-line (*p* = 0.051) and second-line chemotherapy (*p* = 0.018), with a lower risk of progression (HR = 0.29, 95% CI 0.10–0.86; *p* = 0.026). No differences were found between genders with regard to toxicity. *Conclusions.* In our series, PFS rates following first- and second-line therapies for advanced urothelial carcinoma confirmed that females have a greater risk of progression than males.

## 1. Introduction

The incidence of urothelial cancer in males is three to four times greater than in females [1]. Although the male gender is an independent risk factor [2], females are associated with a higher risk of relapse and progression [3,4]. This gap has been attributed to gender differences in smoking, hepatic metabolism of carcinogens and level of exposure to occupational hazards [4]. Discordant results exist regarding the relationship between estrogen receptor activity and urothelial cancer outgrowth [5,6]. Additionally, women are less likely to receive chemotherapy and radical cystectomy [7] or experience good quality care, which could all contribute to the gender disparities in survival outcome [8,9,10]. Significant delays between presentation with haematuria, suggesting urological malignancy, and diagnosis of bladder cancer occur for both men and women, but time lapses may be longer for women on the assumption of a benign disease [8,9,10]. However, data in the literature are conflicting and burdened by methodological biases in population selection and sampling [11].

Chemotherapy is the treatment of choice for metastatic urothelial carcinoma. Guidelines universally recommend cisplatin-based chemotherapy as first-line treatment for eligible patients, but there is no standard chemotherapy for those unfit for this treatment. Immune checkpoint inhibitors can be administered to progressing patients during or after platinum-based treatment, and vinflunine may be a second-line option when immunotherapy or combination chemotherapy is not feasible [12].

Some evidence shows a greater toxicity and higher clinical response rates after chemotherapy in women [13,14]. Indeed, the female sex appears to be a risk factor for clinically relevant adverse events (AEs) [15], although no significant differences exist in the number of chemotherapy cycles or severe toxic events when comparing male and female patients [16]. In particular, women affected by metastatic urothelial cancer cope with cisplatin-based chemotherapy similarly to men [16].

The aim of the present study was to analyze the impact of gender on chemotherapy efficacy and toxicity in advanced and metastatic urothelial carcinoma.

## 2. Materials and Methods

Between January 2013 and October 2018, 98 patients with advanced urothelial cancer were evaluated in a single institution for first-line and second-line chemotherapy. Th inclusion criteria required that patients had received at least one cycle of chemotherapy. Patients unfit for chemotherapy and eligible for base support care (BSC) alone, or those surgically treated at other urological centres were excluded, leaving 75 consecutive patients for the current study, 18 (24%) females and 57 (76%) males.

The following parameters were analyzed: sex, age, smoking habit, site of primary tumour, pathological stage at diagnosis, histotype, site of metastasis, time between diagnosis and progression, radiological response and drug toxicity. The pathological stage was assigned according to the 2017 TNM classification [17]. Response to therapy was estimated according to the Response Evaluation Criteria in Solid Tumours (RECIST) guidelines (version 1.1). Toxicity assessment was performed employing the National Cancer Institute Common Toxicity Criteria (NCI CTC). All patients were interviewed face-to-face about their smoking habits and followed up until the end of the study period (1 June 2019) or date of death.

The study was conducted in accordance with the guidelines of the Declaration of Helsinki and approved by the Local Ethical Committee (Prot. CEAVC_18094). Informed consent was obtained from all subjects involved in the study.

Survival analyses were performed by the Kaplan–Meier method. Observation time for OS started on T1 (date of diagnosis) and ended on T2 (date of last follow-up for living patients, or date of death). PFS was defined as the period from the start date of chemotherapy to the date of disease progression. The log-rank test was used to assess differences in OS and PFS according to selected individual parameters. Through univariate Cox regression analysis, the impact of each parameter on OS and PFS was evaluated. The risk of death or progression (HR, hazard ratio) was calculated with 95% confidence intervals (CI). In the case of multiple significant parameters in the univariate model, multivariate regression analysis was carried out to underline the effect of each parameter adjusted for the others present in the model. The gender distribution of selected categorical parameters was calculated using the Fisher exact test (for two-level parameters) or the chi-square test for trend (for parameters with more than two levels). For continuous variables, the Mann–Whitney U test was employed as appropriate. A *p*-value of <0.05 was considered to be statistically significant.

Due to lack of data, statistical analysis was performed only on 66 of the 71 patients receiving platinum-based chemotherapy for first-line treatment and on the largest sample of patients administered a second-line chemotherapy (15 patients on vinflunine).

## 3. Results

### 3.1. Patient Characteristics

The patients included in the study had undergone primary surgical treatment for urothelial carcinoma of the urinary bladder (67 patients, 89.3%) or the upper urinary tract (8 patients, 10.7%). Table 1 summarizes the distribution of the individual characteristics according to gender. No statistically significant differences were found except for smoking, with a prevalence of current smokers among women (*p* = 0.029).

Of the 75 patients, 69 (92%) had conventional urothelial carcinoma and 6 (8%) showed uncommon variants of urothelial carcinoma, including micropapillary, nested, plasmacytoid and sarcomatoid. There were no gender-related differences in tumour histotype (*p* = 0.63).

At the time of chemotherapy, all patients were affected by stage IV disease. Overall, 44 (58.7%) patients received first-line treatment alone and 31 (41.3%) received second-line treatment.

### 3.2. Time between Diagnosis and Metastasis Detection

The time interval between date of diagnosis and metastasis detection was analyzed. Although longer for women, the difference was not statistically significant. The mean, standard deviation (SD) values and results of the Mann-Whitney U test were 1.12 ± 1.2 years for women vs. 0.94 ± 1.3 years for men (range: 0.01–4.51 vs. 0.01–8.28 years; *p* = 0.50).

### 3.3. Overall Survival (OS)

**Figure 1:**
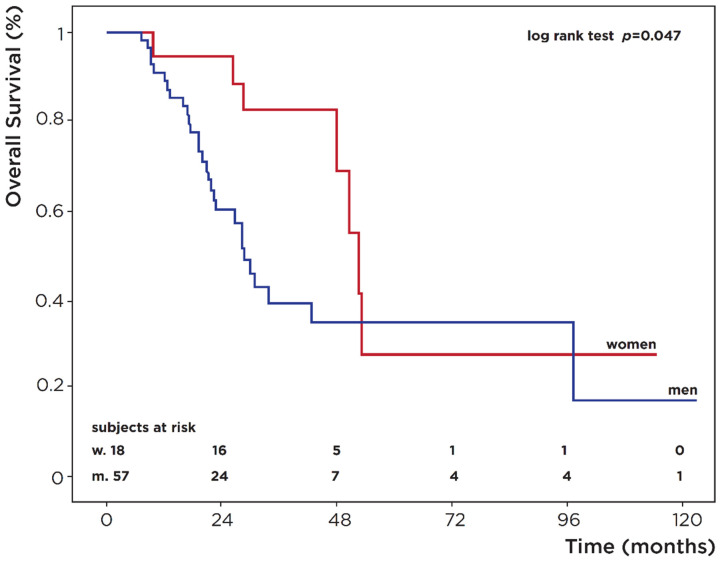
Overall survival (OS) in patients with advanced urothelial cancer.

The mean follow-up interval was 30 months (SD: ±23; range: 7–121; median: 27). Overall, 36 (48%) deaths were recorded (OS: 21.3%) at the 121-month follow-up. OS was significantly higher in females (27.5% vs. 17.4%; *p* = 0.047) (Figure 1, Table 2), and univariate Cox regression analysis confirmed that males had a higher risk of death (HR = 2.28, 95% CI 0.99–5.25), with a borderline statistical significance (*p* = 0.053). None of the other parameters investigated reached statistical significance (Table 2).

### 3.4. Radiological Response and Progression-Free Survival (PFS)

All 75 patients were given first-line treatment: 71 were prescribed platinum-based drugs (54 males and 17 females) and the remaining 4 gemcitabine. Of the 71 patients, 31 (23 males and 8 females) received second-line chemotherapy, 15 on vinflunine (10 males and 5 females) and 16 on carboplatin- or gemcitabine-based regimens.

**Figure 2:**
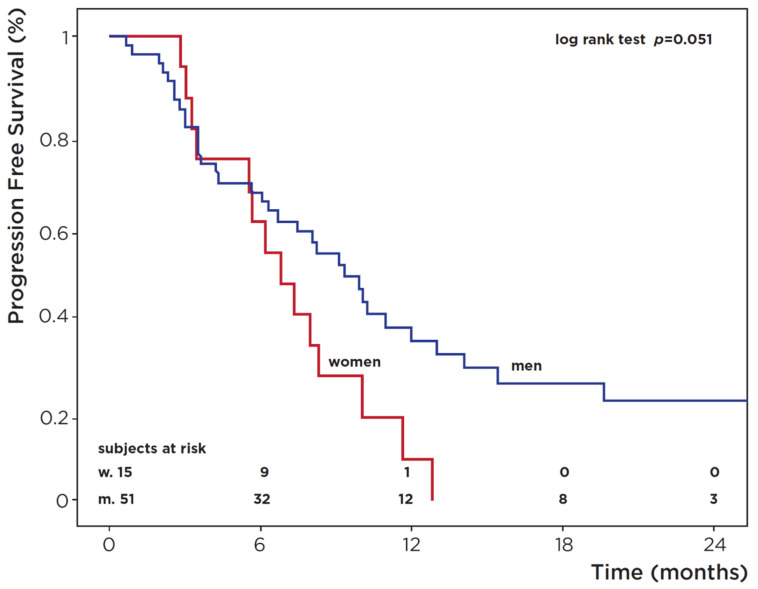
Progression-free survival (PFS) in patients with advanced urothelial cancer.

Treatment efficacy was measured by radiological response and PFS. With regards to radiological response, the chi-square test revealed no difference in distribution by gender for either the first- or second-line therapy (data not shown). Concerning PFS, mean time to first-line progression was 6.7 months (SD: 4.2; median: 6.1). Overall, 48 events (67.6%) were identified at the end of follow-up (approximately 71 months). Kaplan–Meier survival analysis displayed higher PFS rates in men, with borderline statistical significance (21.6% vs. 0%, *p* = 0.051) (Table 3, Figure 2). Significant differences emerged for age (*p* = 0.041), smoking status (*p* = 0.009) and body mass index (BMI, *p* = 0.035), with PFS being lower in patients over 65 and higher in former smokers and overweight patients. Univariate Cox regression analysis showed the effect of advanced age (*p* = 0.044), smoking (*p* = 0.005) and being overweight (*p* = 0.027) on PFS, while the multivariate model retained smoking and being overweight (*p* = 0.009 and *p* = 0.035, respectively) but not age (Table 3).

Regarding second-line chemotherapy, mean time to progression was 4.6 months (SD: 4.7). A total of 12 events (78.9%) were recorded. Kaplan–Meier survival analysis showed a higher PFS in men (*p* = 0.018), and Cox regression analysis confirmed a lower risk of progression for males (HR = 0.29, 95% CI 0.10–0.86; *p* = 0.026). Other parameters had no statistical significance (data not shown).

### 3.5. Toxicity

The majority of the 71 patients on platinum-based regimens experienced AEs (*n* = 69, 97.2%). Grade 1–2 AEs were reported in 49 (69%) patients, and grade 3–4 AEs in 20 (28.2%) patients (Table 4). Grade 1–2 haematological and gastrointestinal toxicities occurred more frequently in males (61.1% and 44.4% vs. 29.4% and 29.4%, respectively), while grade 3–4 haematological AEs were more common in women (41.2% vs. 24.1%), although no significant differences were observed (*p* = 0.07 and *p* = 0.13). Likewise, no significant disparities emerged for renal toxicity and asthenia. However, renal toxicity was mainly reported in males (29.6% vs. 11.8%; *p* = 0.21) and asthenia in females (47% vs. 40.7%; *p* = 0.78); no grade ≥ 3 AEs were recorded.

There was not any significant gender difference in vinflunine-induced toxicities. Any-grade AEs occurred in five (50%) males and four (80%) females (*p* = 0.44). Grade 1–2 haematological toxicity was more frequent in females (40% vs. 20%), but grade 3–4 toxicity rates were similar (20% vs. 30%) (*p* = 0.42). Grade 1–2 gastrointestinal toxicity was recorded in six (60%) males and one (20%) female, with only one female experiencing side-effects of grade 3–4 (*p* = 0.19). Finally, grade 1–2 asthenia was reported in three (60%) females and two (20%) males (*p* = 0.61), while only one male developed grade 1–2 renal events (*p* = 1.0) (Table 4).

## 4. Discussion

Early studies on the prognostic impact of gender in patients with advanced urothelial cancer date back to the turn of the century, but despite the remarkable amount of research carried out, results are still conflicting and ambiguous [4,11,18,19,20,21,22,23,24,25].

Annually, almost 550,000 patients (424,000 men and 125,000 women) are diagnosed with bladder cancer worldwide, and approximately 200,000 patients (148,000 men and 52,000 women) die from this disease [26]. Urothelial bladder cancer is about four times more common in men than in women [26], as in the current study (M:F = 3:1). The neoplasm is age-related [19], and retrospective studies have reported that women are usually older at the time of diagnosis [20,27]. Our analysis, however, found no significant age difference at first diagnosis.

Stratification into prognostic categories might be difficult and is often lacking. Indeed, studies utilizing the Surveillance, Epidemiology, and End Results (SEER) database and other population registries have limitations in disease stage coding [28,29], leading to inaccurate clinical information in 24–70% of patients [21,22]. A recent update from the National Cancer Database has reported a higher percentage of females with advanced stage bladder cancer at diagnosis compared with males [7], as well as an association between female gender and advanced disease [7]. In contrast, our data revealed that stage IV disease at diagnosis was approximately 1.5 times more frequent in males, but this difference was not significant.

Urothelial carcinoma can show conventional morphology or exhibit a distinctively different histological pattern (variants) [30,31]. Recognition of unusual morphological features has diagnostic, prognostic and therapeutic relevance [31,32,33]. The micropapillary variant of urothelial carcinoma comprises 0.6–2.2% of all urothelial cancers, and demonstrates a male predominance with a male to female ratio of 3:1 [2,34]. Similarly, giant cell carcinoma and sarcomatoid carcinoma are more common in men with a ratio of approximately 3:1 [34]. In the present study, there were no significant differences in tumour histology by gender.

Smoking has been recognized as the major risk factor for the development of urothelial carcinoma, with population-attributable risk approaching 50% [35]. Twenty years after giving up smoking, ex-smokers still have an increased risk [36]. In our series, urothelial carcinoma was strongly associated with smoking in 94% of males and 79% of females. Along with multifocality and early recurrences, smoking status can also influence PFS in both non-muscle-invasive and muscle-invasive bladder cancer patients [37,38].

The impact of gender on OS is unclear [4,11,18,19,20,21,22,23,24,25]. While some studies reported overlapping outcomes between genders [19,20], others suggested that the female sex is associated with worse prognosis [21,22]. According to Andreassen et al., women have a less favourable prognosis within the first two years of diagnosis, after which risk rates appear to be higher in men [23]. In addition, Uhlig et al. found that female patients undergoing radical cystectomy for bladder carcinoma demonstrated worse overall, disease-free and cancer specific survival than males [24]. A subsequent meta-analysis by Uhlig et al. showed that women are at a higher risk of recurrence after local treatment of non-muscle invasive bladder carcinoma compared with men [25].

While the current literature mainly discusses clinical results of radical surgery, few studies have focused on gender and chemotherapy regimens for advanced or metastatic disease. In 2013, Keck et al. evaluated the prognostic relevance of gender in patients treated with a combination of irradiation and chemotherapy, demonstrating on multivariate analysis a higher cancer-specific mortality in females (HR = 2.40, *p* < 0.001) [39].

In our series, male patients had lower OS rates (17.4% vs. 27.5%; *p* = 0.047) and higher odds of death than their female counterparts (HR = 2.28, 95% CI 0.99–5.25; *p* = 0.053). Regarding PFS after first- and second-line treatment, our data were in line with the literature, showing the greater risk of progression in women [24,25]. No statistically significant gender difference in the percentage of drug-related AEs emerged. Females experienced greater toxicity (grade 3–4 AEs) with platinum-containing chemotherapy, while grade 3–4 AEs were more common in males after vinflunine treatment.

Limitations of this work, mainly inherent to the retrospective study design, must be acknowledged. An additional limitation was the small sample size of the study population and lack of multi-institution involvement.

## 5. Conclusions

Our analysis revealed gender differences in the oncological outcome of patients with advanced urothelial cancer. Both after first- and second-line treatment, women were at a greater risk of progression, but showed higher OS rates and a lower risk of death. This monocentric and retrospective experience suggested that chemotherapy response might vary significantly between males and females, paving the way for future research in the field of personalised medicine. However, multicentric prospective studies that accurately select the sample population are mandatory.

## Figures and Tables

**Table 1:** Characteristics of 75 patients treated with chemotherapy between 2013 and 2018.

Characteristic	Total (*n* = 75) *n* (%)	Males (*n* = 57) *n* (%)	Females (*n* = 18) *n* (%)	*p*-Value °
Ages at diagnosis, years median (range)	67 (40–85)	67 (40–85)	67 (51–80)	0.22
Stage at diagnosis *				
II	8 (11.4)	6 (11.5)	2 (11.2)	
III	21 (30.0)	13 (25.0)	8 (44.4)	0.29
IV	41 (58.6)	33 (63.5)	8 (44.4)	
Site of primary tumour				
Bladder	67 (89.3)	51 (89.5)	16 (88.9)	1.0
Upper urinary tract	8 (10.7)	6 (10.5)	2 (11.1)	
Histotype				
Conventional	69 (92.0)	53 (93.0)	16 (88.9)	0.63
Variants	6 (8.0)	4 (7.0)	2 (11.1)	
Site of metastasis				
Lung	13 (17.3)	10 (17.5)	3 (16.7)	
Liver	2 (2.7)	2 (3.5)	0 (0)	0.95
Bone	9 (12.0)	7 (12.3)	2 (11.1)	
Other	16 (21.3)	12 (21.1)	4 (22.2)	
Multiple	35 (46.7)	26 (45.6)	9 (50.0)	
Smoking *				
Current	12 (19.4)	7 (14.6)	5 (35.7)	
Never	6 (9.6)	3 (6.3)	3 (21.4)	**0.029**
Former	44 (71.0)	38 (79.1)	6 (42.9)	

* some data are missing; ° from chi-square or Mann–Whitney U test, as appropriate; *p*-values in bold denote statistical significance.

**Table 2:** Kaplan–Meier OS analysis at the end of follow-up (at 120 months).

Overall Survival Analysis				
Characteristic	Pts at Start	Deaths	%OS	*p*-Value °
Sex				
Female	18	7	27.5	
Male	57	29	17.4	**0.047**
Age diagnosis				
≤65	28	11		
>65	47	25	-	0.16
Smoking *				
Current	12	4		
Never	6	6	-	0.055
Former	44	18		
Stage *				
II	8	5		
III	21	9	-	0.24
IV	41	20		
Histology				
Conventional	69	31	-	
Variants	6	5		0.084
Site				
Bladder	67	30	-	0.64
UUTt	8	6		
CHT adjuvant				
No	66	29	-	0.17
Yes	9	7		
CHT I line				
Platinum	71	35	-	0.25
Other	4	1		
CHT II line				
Vinflunine	15	7		
Other	16	11	-	0.64
No	44	18		
BMI				
18.5–24.9	40	21		
25–29.9	28	11	-	0.67
>30	7	4		
Total	75	36	21.3	

* some data are missing; ° *p*-value from log rank test; *p*-values in bold denote statistical significance; OS: overall survival; UUT: upper urinary tract; CHT: chemotherapy; BMI: body mass index.

**Table 3:** Kaplan–Meier PFS survival analysis at approximately 70 months. HR and 95% CI from Cox Regression analysis (uni- and multivariate).

Progression-Free Survival (PFS)					Cox Regression Analysis	
					Univariate	Multivariate
Characteristic	Pts at Start	PD	%PFS	*p*-Value °	HR (95% CI) *p*-Value	HR (95% CI) *p*-Value
Sex						
Female	15	14	0		1	-
Male	51	34	21.6	0.051	0.53 (0.28–1.02) *p* = 0.06	
Age diagnosis						
≤65	22	14	27.6		1	1
>65	44	34	10	**0.041**	1.93 (1.02–3.67) ***p* = 0.044**	1.28 (0.62–2.62) *p* = 0.50
Smoking *						
Current	10	7	26.7		1	1
Never	5	5	0	**0.009**	1.51 (0.65–3.52) *p* = 0.39	1.53 (0.64–3.66) *p* = 0.34
Former	41	27	20.7		4.35 (1.57–12.1) ***p* = 0.005**	4.11 (1.43–11.8) ***p* = 0.009**
Stage *						
II	8	7				
III	17	12	-	0.86	-	-
IV	37	27				
Histology						
Conventional	62	45	-	0.94	-	-
Variants	4	3				
Site						
Bladder	59	42	-	0.74	-	-
UUT	7	6				
BMI						
18.5–24.9	36	28	12.9		1	1
25–29.9	15	15	23.7	**0.035**	0.49 (0.26–0.92) ***p* = 0.027**	0.47 (0.23–0.95) ***p* = 0.035**
>30	5	5	0		1.40 (0.54–3.68) *p* = 0.49	0.97 (0.32–2.93) *p* = 0.96
Total	66	48	16.3			

* some data are missing; ° *p*-value from log rank test; *p*-values in bold denote statistical significance; PD: progression disease; PFS: progression-free survival; HR: hazard ratio; CI: confidence interval; UUT: upper urinary tract; BMI: body mass index.

**Table 4:** Toxicity to treatment with platinum-based chemotherapy and vinflunine *.

	Platinum-Based CHT			Vinflunine		
Toxicity	Females, *n* (%)	Males, *n* (%)	*p* Value	Females, *n* (%)	Males, *n* (%)	*p* Value
Any AEs			0.23			0.44
G1–2	9 (52.9)	40 (74.1)		3 (60)	3 (30)	
G3–4	7 (41.2)	13 (24.1)		1 (20)	2 (20)	
Haematological			0.07			0.42
G1–2	5 (29.4)	33 (61.1)		2 (40)	2 (20)	
G3–4	7 (41.2)	13 (24.1)		1 (20)	3 (30)	
Gastrointestinal			0.13			0.19
G1–2	5 (29.4)	24 (44.4)		1 (20)	6 (60)	
G3–4	0 (0)	0 (0)		1 (20)	0 (0)	
Renal			0.21			1.0
G1–2	2 (11.8)	16 (29.6)		0 (0)	1 (10)	
G3–4	0 (0)	0 (0)		0 (0)	0 (0)	
Asthenia			0.78			0.61
G1–2	8 (47.0)	22 (40.7)		3 (60)	2 (20)	
G3–4	0 (0)	0 (0)		0 (0)	0 (0)	

* a total of 71 patients were treated with platinum-based chemotherapy (17 females, 54 males) and 15 with vinflunine (10 females, 10 males). CHT: chemotherapy; AEs: adverse events; G: grade; *n*: number.

## Data Availability

The data used to support the findings of this study are available from the corresponding author upon request.
